# Impact of the COVID-19 Pandemic on New Long-term Care Insurance Applications and All-cause Mortality in Older Adults in a Japanese Metropolitan Cohort: An Interrupted Time-series Analysis

**DOI:** 10.2188/jea.JE20240464

**Published:** 2025-10-05

**Authors:** Satoshi Seino, Toshiki Hata, Hiroki Mori, Shoji Shinkai, Yoshinori Fujiwara, Erika Kobayashi

**Affiliations:** 1 Research Team for Social Participation and Healthy Aging, Tokyo Metropolitan Institute for Geriatrics and Gerontology, Tokyo, Japan; 2 Institute of Well-Being, Yamagata University, Yamagata, Japan; 3 School of Food and Nutritional Sciences, The University of Shizuoka, Shizuoka, Japan; 4 Department of Nutrition Sciences, Kagawa Nutrition University, Saitama, Japan; 5 Tokyo Metropolitan Institute for Geriatrics and Gerontology, Tokyo, Japan

**Keywords:** COVID-19, pandemic, long-term care, mortality, older adults, interrupted time-series analysis

## Abstract

**Background:**

New long-term care insurance (LTCI) certifications and mortality are key outcomes in cohort studies involving older adults; however, the coronavirus disease 2019 (COVID-19)’s comprehensive impacts on these outcomes remain underexplored. We examined the pandemic’s impact on new LTCI applications and all-cause mortality in a metropolitan cohort.

**Methods:**

In 2016, 15,500 individuals aged 65–84 years were randomly selected through stratified sampling from Ota City, Tokyo. LTCI and death records were tracked through December 2023; the monthly LTCI applications and all-cause deaths per 10,000 people were calculated. The COVID-19 pandemic period was defined as beginning in March 2020, after the World Health Organization Director-General characterized the situation as a pandemic on March 11, 2020. Interrupted time-series segmented regression analysis was used to compare trends pre- (January 2018–February 2020) and post-pandemic onset (March 2020–December 2023).

**Results:**

From January 2018 to December 2023, 4,083 new LTCI applications and 2,457 deaths were recorded. New monthly LTCI applications showed a modest upward trend pre-pandemic (0.4 per 10,000 people; 95% confidence interval [CI], 0.1–0.8), declined sharply at the pandemic’s onset (−9.6 per 10,000 people; 95% CI, −16.0 to −3.2), and subsequently increased at a higher rate than pre-pandemic levels (0.8 per 10,000 people; 95% CI, 0.6–1.0). Monthly all-cause deaths remained stable before and immediately after the pandemic’s onset but rose slightly in the post-pandemic period (0.3 per 10,000 people per month; 95% CI, 0.2–0.5).

**Conclusion:**

The COVID-19 pandemic influenced both new LTCI applications and all-cause mortality in this study. These impacts should be carefully considered in cohort studies examining these outcomes.

## INTRODUCTION

New long-term care insurance (LTCI) certifications^1^^,^^2^ and mortality are critical health outcomes frequently analyzed in epidemiological studies involving older adults. In Japan, the coronavirus disease 2019 (COVID-19) pandemic caused a decline in physical activity^3^ and an increase in “pandemic-associated-frailty”^3^^,^^4^ among older adults, raising concerns about a potential rise in LTCI certifications and mortality. Therefore, assessing the pandemic’s impact on these outcomes is crucial for ongoing cohort studies.

Previous studies have suggested that while the COVID-19 pandemic did not lead to a significant nationwide increase in LTCI certification rates in Japan,^5^ excess mortality was observed.^6^^–^^8^ However, these impacts may have varied across regions depending on local infection levels. For example, during the early stages of the pandemic, densely populated urban areas with a higher number of infections reported a negative impact on frailty among older adults.^9^

Our previous report^10^ identified a trade-off between increasing severe acute respiratory syndrome coronavirus 2 (SARS-CoV-2) infections and declining LTCI applications during the first four waves of the pandemic. However, the broader and longer-term impacts of COVID-19 on new LTCI applications and all-cause mortality remain unclear.

This report offers a comprehensive analysis of the pandemic’s impacts on these outcomes within our cohort, providing new insights into the long-term implications of COVID-19 on the health and care needs of older adults.

## METHODS

### Study population

This study utilized data from a community-wide intervention study initiated in 2016 in Ota City, Tokyo, Japan, with the goal of preventing frailty.^11^^,^^12^ Details of the study design and participant characteristics have been reported previously.^11^ In summary, 15,500 residents aged 65–84 years, who were not certified as requiring LTCI as of June 1, 2016, were randomly selected from all 18 administrative districts of Ota City through stratified sampling based on age group (aged 65–74 and 75–84 years) and sex.^11^

The Ethical Committee of the Tokyo Metropolitan Institute for Geriatrics and Gerontology approved this study on June 7, 2018 (reference number: 22). The study was conducted using an opt-out method, with information about the study declared publicly to provide participants the opportunity to decline participation.

### Measurements

#### LTCI applications and all-cause mortality

We obtained LTCI and all-cause mortality data from the local government till December 31, 2023.

LTCI applications were tracked using the mandatory Japanese LTCI system database, which covers all adults aged ≥40 years. The certification process involves a standardized, multistep evaluation.^1^ Applications can be initiated by older adults, their families, or caregivers. A trained local government official conducts a home visit to assess the applicant’s physical and mental condition using a 74-item standardized questionnaire. A medical opinion is submitted by the applicant’s attending physician or a designated physician based on a standardized manual.^13^^,^^14^ A computer-based system performs an initial assessment, and the final decision is made by the Municipal Certification Committee, which assigns care needs to one of the seven levels: two support levels (1–2) and five care levels (1–5), with level 5 indicating the highest level of functional disability. In our cohort, over 99% of LTCI applicants were certified. Due to the approximately 1-month lag between application and certification, the analysis focused on the application date.

All deaths were identified through local registries linked to the Japanese National Vital Statistics System.

Following a previous methodology,^10^ we calculated the monthly new LTCI applications and all-cause deaths per 10,000 individuals from January 2018 to December 2023.

#### Daily counts of SARS-CoV-2 infections and COVID-19-related deaths in Tokyo

To align monthly LTCI application and death data with pandemic trends, we obtained daily counts of new SARS-CoV-2 infections and COVID-19-related deaths in Tokyo through May 7, 2023 (immediately before the category of COVID-19 was changed to a Category V Infectious Disease under the Infectious Disease Control Law) from the Ministry of Health, Labour and Welfare’s open data portal.^15^

### Statistical analyses

We defined the COVID-19 pandemic period as beginning in March 2020, after the World Health Organization Director-General characterized the situation as a pandemic on March 11, 2020.^16^ To compare trends in the number of LTCI applications and all-cause deaths during the 2.2 years before the pandemic (January 2018 to February 2020) and the 3.8 years following its onset (March 2020 to December 2023), we applied an interrupted time-series analysis using segmented regression.^17^ Changes in level and slope for LTCI applications and all-cause mortality were estimated. Seasonality was accounted for by including Fourier terms with two harmonics (sine and cosine pairs) in the model.

Data were analyzed using Stata 18.0 (StataCorp, Colege Station, TX, USA), and an α of 0.05 indicated statistical significance.

## RESULTS

During the observation period (January 2018 to December 2023), 4,083 new LTCI applications and 2,457 deaths were recorded.

**Figure 1.**
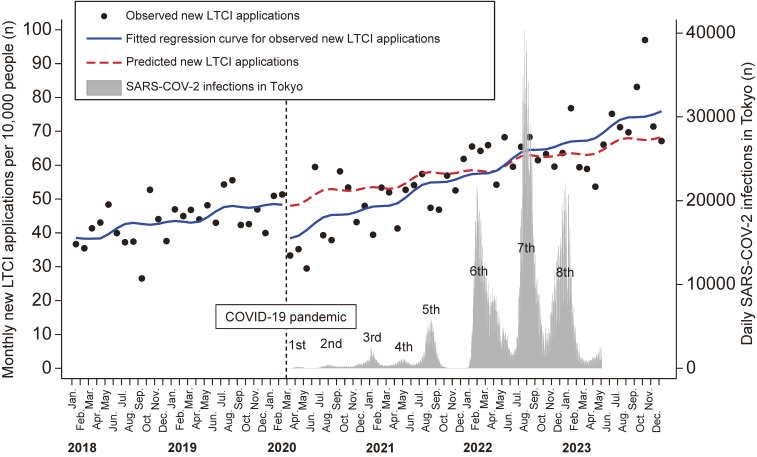
Monthly new LTCI applications per 10,000 people in the Ota City cohort and daily SARS-CoV-2 infections in Tokyo. The solid line represents the regression curve, adjusted for seasonality, for new LTCI applications before the pandemic (January 2018 to February 2020) and during the pandemic (March 2020 to December 2023). The dotted red line indicates predicted values based on the pre-pandemic regression model. The grey bars depict daily SARS-CoV-2 infections in Tokyo. COVID-19, coronavirus disease 2019; LTCI, long-term care insurance; SARS-CoV-2, Severe acute respiratory syndrome coronavirus 2.

Figure 1 shows the monthly number of new LTCI applications per 10,000 people in the Ota City cohort alongside daily SARS-CoV-2 infections in Tokyo. Before the pandemic, new LTCI applications showed a slight but significant increasing trend (0.4 per 10,000 people per month; 95% confidence interval [CI], 0.1–0.8). At the pandemic’s onset, a significant immediate decrease was observed in new LTCI applications (−9.6 per 10,000 people per month; 95% CI, −16.0 to −3.2). Subsequently, new LTCI applications increased at a significantly higher rate than before the pandemic (0.8 per 10,000 people per month; 95% CI, 0.6–1.0).

**Figure 2.**
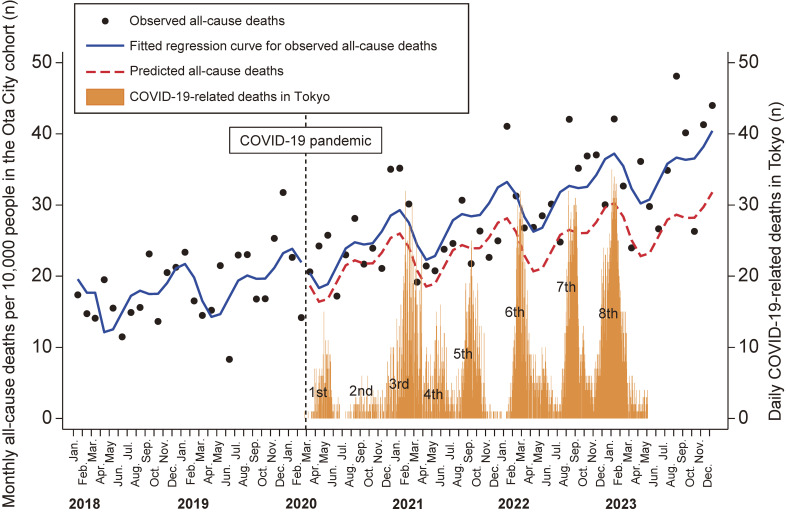
Monthly all-cause deaths per 10,000 people in the Ota City cohort and daily COVID-19-related deaths in Tokyo. The solid line displays the regression curve, adjusted for seasonality, for all-cause deaths before (January 2018 to February 2020) and during the pandemic (March 2020 to December 2023). The dotted red line represents predicted values based on the pre-pandemic regression model. The orange bars indicate daily COVID-19-related deaths in Tokyo. COVID-19, coronavirus disease 2019.

Figure 2 shows monthly all-cause deaths per 10,000 people in the Ota City cohort and daily COVID-19-related deaths in Tokyo. The number of all-cause deaths remained stable before the COVID-19 pandemic (0.2 per 10,000 people per month; 95% CI, −0.1 to 0.5) and did not change significantly immediately after the pandemic began (1.8 per 10,000 people per month; 95% CI, −3.7 to 7.2). However, post-pandemic onset, the number of all-cause deaths exhibited a slight but significant increasing trend compared to the pre-pandemic period (0.3 per 10,000 people per month; 95% CI, 0.2–0.5).

## DISCUSSION

New LTCI applications in our cohort showed a slight but significant upward trend before the pandemic, experienced a sharp immediate decline at the onset of the COVID-19 pandemic, and then increased at a significantly higher rate than the pre-pandemic period. Meanwhile, monthly all-cause deaths in our cohort remained stable before and immediately after the pandemic’s onset but exhibited a slight yet significant upward trend in the post-pandemic period. The COVID-19 pandemic had a non-negligible impact on both new LTCI applications and all-cause mortality in our cohort.

A previous report^5^ demonstrated that the national LTCI certification rate temporarily declined at the onset of the COVID-19 pandemic but later returned to pre-pandemic trends by January 2024, with only minimal overall impact. In contrast, our metropolitan cohort exhibited a more pronounced reduction in the number of applications, followed by a steeper upward trend, suggesting a greater impact of the pandemic in urban areas with higher infection rates. Because new LTCI applications in Ota City continued as usual during the COVID-19 pandemic, the marked decline in applications during the pandemic’s early stages may be attributed to users’ perspectives, including heightened concerns about infection during the LTCI application and certification procedures, along with increased family support facilitated by teleworking arrangements. The subsequent upward trend in new LTCI applications, which eventually exceeded pre-pandemic projections, likely reflects the ongoing but incomplete recovery of physical activity and/or social engagement.^18^^,^^19^

In our cohort, a gradual increase in all-cause mortality, likely reflecting excess deaths, was observed and aligned with nationwide trends.^6^^–^^8^ In Japan, while the impact of COVID-19 on life expectancy was limited in 2020, a notable shift in mortality trends emerged in 2021, with all-cause age-standardized mortality rates rising by 2% compared to that in 2020.^7^ Between 2021 and 2022, this increase accelerated to approximately 6%, marking the largest rise in half a century and driven primarily by significant increases in deaths attributed to COVID-19, senility, and heart disease.^8^ Given the recent upward trend in LTCI applications, continued monitoring of cause-specific mortality trends is crucial to understanding the long-term impacts of the pandemic.

Our findings have important implications for public health policy. First, the marked decline in new LTCI applications during the early stages of the pandemic underscored the need for contingency planning to maintain access to LTCI services during public health emergencies. As concerns about infection associated with home visit assessments after applications likely contributed to this decline,^20^ implementing more flexible LTCI certification procedures under crisis conditions—including digital application systems or remote evaluations—could help mitigate service disruptions in future crises.

Second, the observed post-pandemic surge in LTCI applications highlighted the importance of early preventive interventions during public health emergencies. It is essential to develop preventive programs that are feasible even under crisis conditions and to foster strong social connections that support the timely resumption of physical and social activities—particularly among frail older adults—once such conditions have been resolved. These efforts are expected to contribute to both the improvement of older adults’ well-being and help mitigate the long-term burden on the LTCI system.

A key strength of this report is its comprehensive analysis of the impact of COVID-19 on new LTCI applications and all-cause mortality within a cohort of older adults in a densely populated Tokyo ward, one of Japan’s most heavily affected areas. However, the sharp rise in new LTCI applications since 2022, coupled with a relatively modest increase in mortality rates,^8^ reflects a metropolitan-specific trend that may not be applicable to other regions. This represents a notable limitation of this study. Furthermore, the baseline upper age limit of 84 years may have resulted in an underestimation of excess mortality, especially related to COVID-19 and senility. Future studies should examine trends across municipalities of different urban scales to better understand the broader impacts of the pandemic on new LTCI certifications and mortality.

### Conclusion

The COVID-19 pandemic significantly impacted new LTCI applications and all-cause mortality in our metropolitan cohort of older adults. Specifically, new LTCI applications initially showed a modest upward trend, sharply declined at the pandemic’s onset, and then increased at a higher rate than pre-pandemic levels, while monthly deaths remained stable initially but rose slightly in the post-pandemic period. These impacts may vary regionally. Cohort studies using new LTCI certifications or mortality as outcomes during the pandemic should examine the potential impact of the pandemic on these outcomes and, if necessary, account for this impact in their analyses and interpretations.
